# Physical activity and the risk of sudden cardiac death: a systematic review and meta-analysis of prospective studies

**DOI:** 10.1186/s12872-020-01531-z

**Published:** 2020-07-06

**Authors:** Dagfinn Aune, Sabrina Schlesinger, Mark Hamer, Teresa Norat, Elio Riboli

**Affiliations:** 1 Department of Epidemiology and Biostatistics, School of Public Health, Imperial College, St. Mary’s Campus, Norfolk Place, Paddington, London, W2 1PG UK; 2 Department of Nutrition, Bjørknes University College, Oslo, Norway; 3 Department of Endocrinology, Morbid Obesity and Preventive Medicine, Oslo University Hospital Ullevål, Oslo, Norway; 4 Institute for Biometry and Epidemiology, German Diabetes Center, Leibniz Institute for Diabetes Research at the Heinrich-Heine-University Düsseldorf, Düsseldorf, Germany; 5 Institute Sport Exercise & Health, Division Surgery Interventional Science, Institute Sport Exercise & Health, Division Surgery Interventional Science, Loughborough, UK; 6 Department of Epidemiology and Public Health, University College London, London, UK

**Keywords:** Physical activity, Cardiorespiratory fitness, Sudden cardiac death, Systematic review, Meta-analysis

## Abstract

**Background:**

Physical activity has been associated with a significant reduction in risk of sudden cardiac death in epidemiological studies, however, the strength of the association needs clarification. We conducted a systematic review and meta-analysis to summarize the available data from population-based prospective studies.

**Methods:**

PubMed and Embase databases were searched for studies of physical activity and sudden cardiac death from inception to March 26th 2019. Prospective studies reporting adjusted relative risk (RR) estimates and 95% confidence intervals (CIs) of sudden cardiac death associated with physical activity were included. A random effects model was used to estimate summary RRs (95% CIs).

**Results:**

Thirteen prospective studies were included in the systematic review. Eight prospective studies with 1193 sudden cardiac deaths among 136,298 participants were included in the meta-analysis of physical activity and sudden cardiac death and the summary RR for highest vs. lowest level of physical activity was 0.52 (95% CI: 0.45–0.60, I^2^ = 0%, p_heterogeneity_ = 0.72). The association was similar in men and women and among American and European studies. In the dose-response analysis the summary RR was 0.68 (95% CI: 0.55–0.86, I^2^ = 44%, *n* = 3) per 20 MET-hours/week. Although the test for nonlinearity was not significant, p_nonlinearity_ = 0.18, there was no further reduction in risk beyond 20–25 MET-hours/week. The summary RR was 0.58 (95% CI: 0.41–0.81, I^2^ = 0%, p_heterogeneity_ = 0.65, *n* = 2) for the highest vs. the lowest level of cardiorespiratory fitness.

**Conclusion:**

This meta-analysis suggest that a high compared to a low level of physical activity may reduce the risk of sudden cardiac death in the general population. Further studies are needed to clarify the dose-response relationship between specific subtypes and intensities of physical activity in relation to sudden cardiac death.

## Background

Cardiovascular disease accounted for 17.8 million deaths worldwide in 2017, making it the leading cause of death globally [1]. Sudden cardiac deaths account for 40–50% of all cardiovascular deaths and 15–20% of all deaths [2] and most of these are due to ventricular tachyarrhythmias [3]. Approximately 350,000 sudden cardiac deaths occur annually in the US [4]. Cardiac arrests usually present without warning signs or symptoms and usually have a fatal outcome [5, 6]. Although preventive efforts have focused on using cardioverter-defibrillators in the highest risk groups, the majority of sudden cardiac deaths occur in the general population and in persons without diagnosed cardiac disease [7, 8]. Population-wide strategies may therefore be a more promising approach for primary prevention of sudden cardiac deaths, however, less is known about risk factors for sudden cardiac deaths than for ischemic heart disease and stroke. Some suspected or established risk factors for sudden cardiac deaths include overweight and obesity, diabetes, high blood pressure, high resting heart rate, smoking, prevalent coronary heart disease, and male sex [9–15].

While physical activity is an established protective factor for coronary heart disease, stroke, and heart failure [16, 17], the association between physical activity and sudden cardiac death has been studied less frequently, and has to our knowledge not been summarized in a meta-analysis previously. Sudden cardiac death has also not yet been included as an outcome in the comparative risk assessments of the Global Burden of Disease project [18], which could potentially lead to underestimates of the mortality burden due to low physical activity, although coronary heart disease and sudden cardiac death are closely linked. Several prospective studies from the general population have reported a reduced risk of sudden cardiac death with moderate or high versus low levels of physical activity [9, 12, 19], however, not all studies found a statistically significant association [20, 21] and some variation in the size of the observed associations have been observed with relative risks ranging from 0.40 to 0.67 [9, 12, 19–21]. A few studies also reported inverse associations between cardiorespiratory fitness and sudden cardiac death [22, 23]. Given the relatively limited number of established risk factors for sudden cardiac death it is of major public health importance to clarify whether there is an association between physical activity and sudden cardiac death and to define the strength and shape of the dose-response relationship more precisely. This is important for primary prevention, comparative risk assessment purposes, and identification of knowledge gaps and further areas of research. We therefore conducted a systematic review and meta-analysis of population-based prospective studies on physical activity and cardiorespiratory fitness and the risk of sudden cardiac death.

## Methods

### Search strategy

Pubmed, and Embase were searched for relevant studies up to July 20th 2017 and the search was updated to 26th of March 2019. The search strategy is found in the Supplementary Text. PRISMA criteria for reporting of meta-analyses was followed [24]. We also screened the reference lists of the included publications for any additional relevant studies.

### Study selection

Published retrospective and prospective cohort studies and nested case-control studies within cohorts that investigated the association between physical activity and the risk of sudden cardiac death in adults from the general population were eligible for inclusion (only prospective cohort studies were identified). Studies on athletes and specific patient populations were excluded. Adjusted relative risk (RR) estimates and 95% confidence intervals (CIs) were required for inclusion. The excluded studies and reasons for exclusion is found in Supplementary Table 1. DA conducted the first step of the literature screening and screened all the references, while DA and SS conducted the second step of the literature screening of the 72 selected articles that were potentially relevant in duplicate.

### Data extraction

The following data were extracted: The first author’s name, publication year, country where the study was conducted, study period, number of cases and participants, subgroup, RRs and 95% CIs for high vs. low physical activity and sudden cardiac death and covariates adjusted for in the analysis. Data were extracted by DA and checked for accuracy by SS.

### Statistical methods

Random effects models were used to estimate summary RRs (95% CIs) of sudden cardiac death for the highest compared to the lowest level of physical activity and per 20 MET-hours/week and for cardiorespiratory fitness [25]. The average of the natural logarithm of the RRs was estimated and the RR from each study was weighted by the inverse of its variance. For the linear dose-response analysis we used the method by Greenland and Longnecker to estimate linear trends and (CIs) across categories of physical activity [26]. For studies that reported physical activity by ranges of activity we calculated the average of the upper and lower cut-off value to get an estimate of the midpoint for each category. For studies with open-ended extreme categories we used the width of the adjacent category to estimate an upper and lower cut-off for the highest and lowest category, respectively. Nonlinear dose-response analyses were conducted using restricted cubic splines with knots at 10, 50 and 90% percentiles of the distribution of physical activity, which was combined using multivariate meta-analysis [27, 28]. For one study which reported on cardiorespiratory fitness in ml O_2_/kg/min we converted the values to METs by dividing by 3.5 [29].

Q and I^2^ statistics were used to evaluate heterogeneity [30]. I^2^ is a measure of how much of the heterogeneity that is due to between study variation rather than chance. I^2^-values of around 25, 50 and 75% indicates low, moderate and high heterogeneity respectively. We conducted main analyses (all studies combined) and stratified by study characteristics such as sample size, number of cases, geographic location, study quality and by adjustment for confounding factors to clarify whether the results were consistent across various subgroups. Meta-regression analyses were conducted to test for heterogeneity between subgroups. Study quality was assessed using the Newcastle Ottawa scale which rates studies according to selection, comparability and outcome assessment with a score range from 0 to 9 [31].

The statistical analyses were conducted using the software package Stata, version 13.1 software (StataCorp, Texas, US).

## Results

**Fig. 1:**
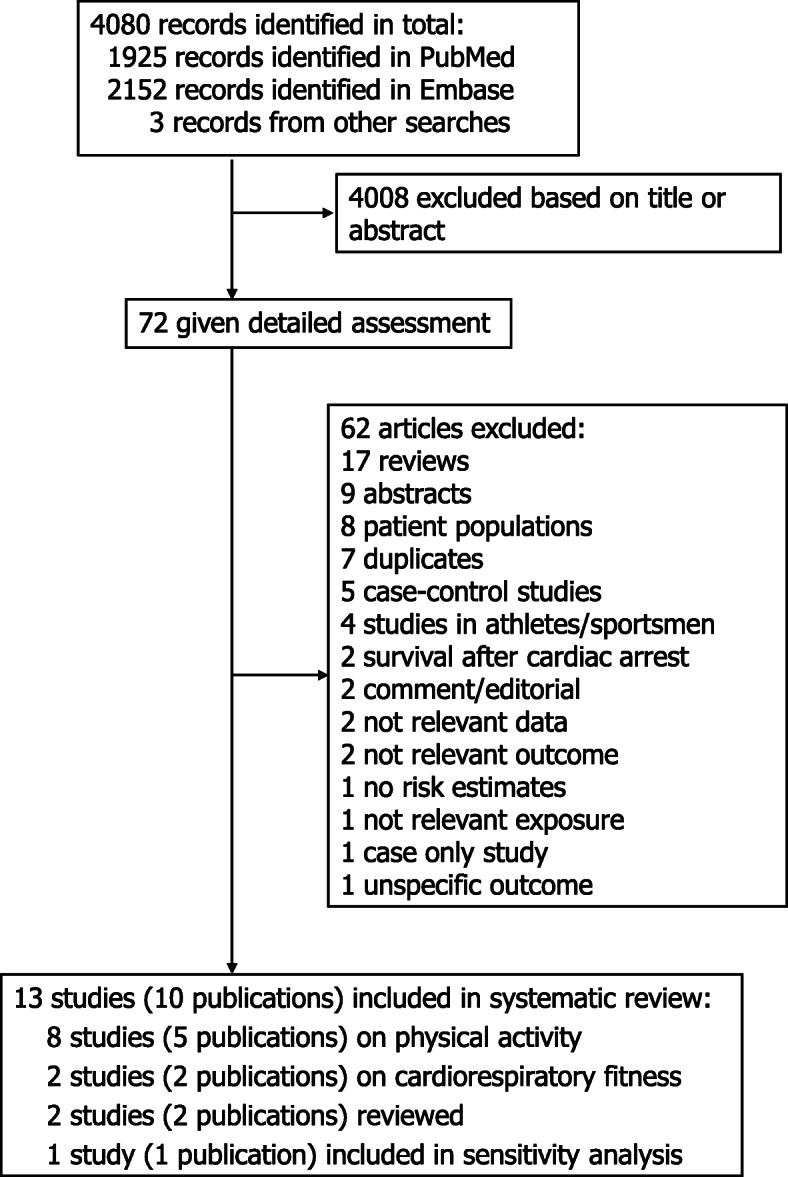
Flow-chart of study selection

**Fig. 2:**
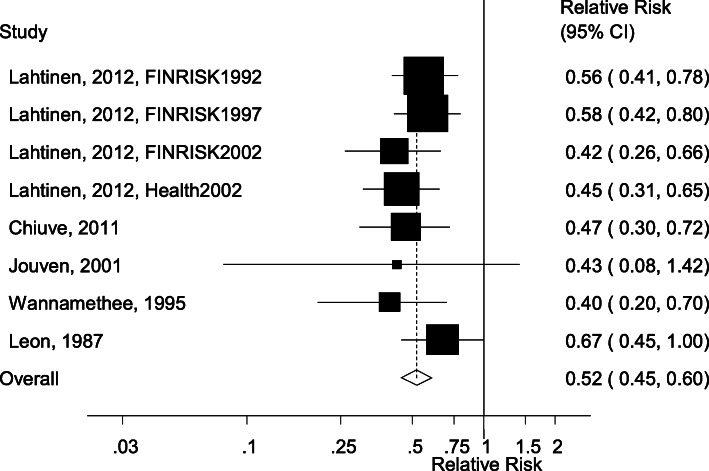
Physical activity and sudden cardiac death

The literature search identified 4080 records in total from which 72 were assessed in more detail and ten publications with data from thirteen prospective cohort studies were included in the systematic review and meta-analysis (Fig. 1). Eight cohort studies (5 publications) [9, 12, 19–21] were included in the meta-analysis of leisure-time physical activity (defined as leisure-time physical activity, exercise or sports) and risk of sudden cardiac death including 1193 sudden cardiac deaths among 136,298 participants (Fig. 2, Table 1). Six of the studies were from Europe and two were from the US and the duration of follow-up ranged from 6 to 26 years. One additional study from the United Kingdom reported on sudden cardiac death and arrhythmias combined and was included in a sensitivity analysis [32]. In addition, two studies on cardiorespiratory fitness were analyzed separately [22, 23], and two studies on vigorous activity [33] and occupational activity [34] were reviewed. The summary RR for the highest vs. lowest leisure-time physical activity was 0.52 (95% CI: 0.45–0.60, I^2^ = 0%, p_heterogeneity_ = 0.72) (Fig. 2). The summary RR ranged from 0.50 (95% CI: 0.42–0.59) when excluding the FINRISK 1997 study [12] to 0.53 (95% CI: 0.45–0.62) when excluding the Health 2002 study [12] (Supplementary Figure 1).

**Table 1:** Prospective studies of physical activity and cardiorespiratory fitness and sudden cardiac death

First author, publication year, country	Study name or description	Study period	Number of participants, number of cases	Type of physical activity, subgroup	Comparison	Relative risk (95% confidence interval)	Adjustment for confounders
Leon AS et al., 1987 [20], USA	The Multiple Risk Factor Intervention Trial	1973–1976 - 1982, 7 years follow-up	12,138 men, age 35–57 years: 143 sudden cardiac deaths	Leisure-time physical activity	1 2 3	1.00 0.64 (0.54–0.96) 0.67 (0.45–1.00)	Age, diastolic blood pressure, total cholesterol, cigarettes per day, treatment group
Wannamethee G et al., 1995 [19], United Kingdom	British Regional Heart Study	1978–1980 - NA, 8 years follow-up	7730 men, age 40–59 years: 117 sudden cardiac deaths	Leisure-time physical activity	None-occasional Light Moderate Moderately vigorous-vigorous	1.0 0.7 (0.5–1.1) 0.6 (0.4–1.1) 0.4 (0.2–0.7)	Age
Jouven X et al., 2001 [21], France	Paris Prospective Study 1	1967–1972 - 1994, 23 years follow-up	7079 men and women, age 42–53 years: 118 sudden deaths	Sports activity	Yes vs. no	0.43 (0.08–1.42)	Age, parental sudden death, parental myocardial infarction, diabetes, tobacco, BMI, resting heart rate, systolic blood pressure, total cholesterol
Chiuve SE et al., 2011 [9], USA	Nurses’ Health Study	1984–2010, 26 years follow-up	81,722 women, age 38–63 years: 321 sudden cardiac deaths	Exercise	< 1 h/wk. 1–1.9 2–3.4 3.5–5.9 ≥6.0	1.00 0.86 (0.60–1.23) 0.84 (0.58–1.21) 0.72 (0.50–1.04) 0.47 (0.30–0.72)	Age, family history of myocardial infarction, menopausal status, current hormone therapy use, diabetes, hypertension, high cholesterol, cancer, coronary heart disease, stroke
Lahtinen AM et al., 2012 [12], Finland	FINRISK 1992	1992–2008, ~ 16 years follow-up	5345 men and women, mean age 44.3 years: 129 sudden cardiac deaths	Leisure-time physical activity	Moderate/high vs. low	0.56 (0.41–0.78)	Age, sex, geographic region, HDL/TC ratio, systolic blood pressure, smoking, diabetes, BMI, prevalent coronary heart disease, QT-prolonging drug, digoxin
Lahtinen AM et al., 2012 [12], Finland	FINRISK 1997	1997–2008, ~ 11 years follow-up	7672 men and women, mean age 48.4 years: 178 sudden cardiac deaths	Leisure-time physical activity	Moderate/high vs. low	0.58 (0.42–0.80)	Age, sex, geographic region, HDL/TC ratio, systolic blood pressure, smoking, diabetes, BMI, prevalent coronary heart disease, QT-prolonging drug, digoxin
Lahtinen AM et al., 2012 [12], Finland	FINRISK 2002	2002–2008, ~ 6 years follow-up	8212 men and women, mean age 48.0 years: 75 sudden cardiac deaths	Leisure-time physical activity	Moderate/high vs. low	0.42 (0.26–0.66)	Age, sex, geographic region, HDL/TC ratio, systolic blood pressure, smoking, diabetes, BMI, prevalent coronary heart disease, QT-prolonging drug, digoxin
Lahtinen AM et al., 2012 [12], Finland	Health 2000	2000–2008, ~ 8 years follow-up	6400 men and women, mean age 53.0 years: 112 sudden cardiac deaths	Leisure-time physical activity	Moderate/high vs. low	0.45 (0.31–0.65)	Age, sex, geographic region, HDL/TC ratio, systolic blood pressure, smoking, diabetes, BMI, prevalent coronary heart disease, QT- prolonging drug, digoxin
Hamer M et al., 2018 [32], United Kingdom	The Health Survey for England and the Scottish Health Surveys	1994, 1995, 1997, 1998, 1999, 2003, 2004, 2006, 2008–2009, 9.4 years follow-up	65,093 men and women, age ≥ 40 years: 70 arrhythmia/ sudden cardiac deaths	Leisure-time physical activity - meeting recommendations Leisure-time physical activity	Inactive Insufficient Sufficient High < 1.64 MET-hours/wk. 1.65–9.37 9.38–19.30 19.31–37.60 > 37.60	1.00 0.48 (0.14–1.20) 0.52 (0.12–2.15) 0.33 (0.10–2.38) 1.00 0.96 (0.53–1.76) 0.68 (0.32–1.43) 0.49 (0.20–1.16) 0.18 (0.04–0.76)	Age, sex, smoking, social occupational group, chronic illnesses, psychological distress
Paffenberger RS et al., 1975, [34] USA	Longshoremen	1951–1961 - 1972, 14.6 years follow-up	6351 men, age 35–74 years: 184 sudden cardiac deaths	Work activity	1 2 3	1.00 0.29 (0.20–0.42) 0.36 (0.22–0.59)	Age
Albert CM et al., 2000 [33], USA	Physicians’ Health Study	1982–1994, 12 years follow-up	21,481 men, age 40–84 years: 109 sudden cardiac deaths	Vigorous exercise	< 1 time/wk. 1/wk. 2–4/wk. ≥5/wk	1.00 1.68 (0.98–2.87) 1.13 (0.69–1.88) 1.36 (0.76–2.43)	Age, assignment to aspirin and beta-carotene treatment, BMI, smoking status, cigarettes per day, diabetes, hypertension, hypercholesterolemia, alcohol, vitamin E, vitamin C, multivitamin use, fish
Jimenez-Pavon D et al., 2016 [22], USA	Aerobics Center Longitudinal Study	1974–2002 - 2003, 14.7 years follow-up	55,456 men and women, mean age 44.2 years: 109 sudden cardiac deaths	Cardiorespiratory fitness	1 2 3 Per 1 MET	1.00 0.56 (0.35–0.90) 0.52 (0.30–0.92) 0.86 (0.77–0.96)	Age, sex, examination year, BMI, smoking, alcohol, diabetes mellitus, hypertension, electrocardiogram showing abnormalities, parental history of cardiovascular disease
Jae SY et al., 2018 [23], Finland	Kuopio Ischaemic Heart Disease Risk Factors Study	1984–1989 - 2014, 22 years follow-up	2357 men, age 42–60 years: 253 sudden cardiac deaths	Cardiorespiratory fitness	< 26.7 ml/kg/min 26.7–33.0 > 33.0	1.00 0.80 (0.58–1.10) 0.61 (0.40–0.92)	Age, smoking, SBP, HDL cholesterol, LDL cholesterol, glucose, diabetes, hypertension, FH - CHD, previous MI, physical activity, BMI

*BMI* Body mass index, *HDL/TC ratio* High-Density Lipoprotein to total cholesterol ratio, *NA* not available

A UK study which reported on sudden cardiac death and arrhythmias combined [32] was included with the eight other studies in a sensitivity analysis [9, 12, 19–21, 32] (*n* = 9 studies, 1263 events and 201,391 participants) and the summary RR was 0.51 (95% CI: 0.44–0.60, I^2^ = 0%). Alternatively, using WHO recommendations for leisure-time physical activity categories from the study by Hamer et al. [32], the summary RR was 0.52 (95% CI: 0.45–0.60, I^2^ = 0%).

**Fig. 3:**
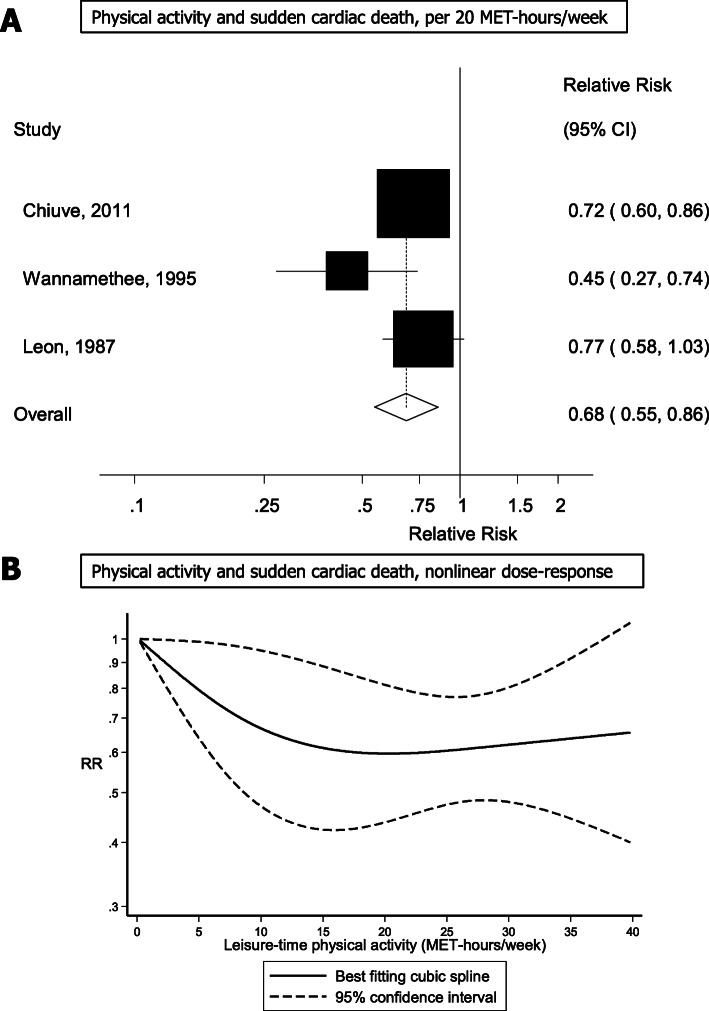
Physical activity and sudden cardiac death, linear and nonlinear dose-response analysis

Three studies [9, 19, 20] were included in the dose-response analysis (581 sudden cardiac deaths, 101,590 participants) and the summary RR per 20 MET-hours/week increase in leisure-time physical activity was 0.68 (95% CI: 0.55–0.86, I^2^ = 44%) (Fig. 3a). The test for nonlinearity was not significant, p_nonlinearity_ = 0.18, however, the dose-response curve appeared largely flat from around 20–25 MET-hours/week and above (Fig. 3b, Supplementary Table 2). The study by Hamer et al. [32] was included in a sensitivity analysis [9, 19, 20, 32] (651 events, 166,683 participants) and the summary RR per 20 MET-hours/week increase in physical activity was 0.66 (95% CI: 0.53–0.81, I^2^ = 40%) (Supplementary Figure 2a). The test for nonlinearity was not significant, p_nonlinearity_ = 0.35, however, again the dose-response curve appeared largely flat from around 20–25 MET-hours/week and above (Fig. 2b, Supplementary Table 2).

**Fig. 4:**
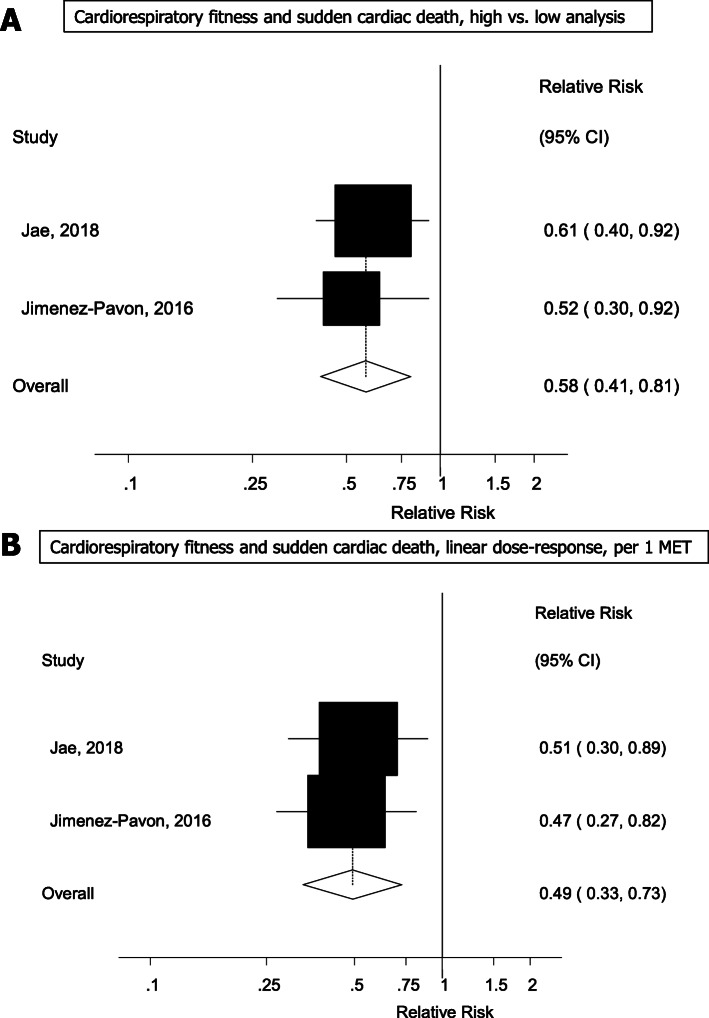
Cardiorespiratory fitness and sudden cardiac death, high vs. low and per 1 MET increase

Two studies [22, 23] were included in the analysis of cardiorespiratory fitness and sudden cardiac death (255 sudden cardiac deaths, 57,824 participants). The summary RR for the highest vs. lowest level of cardiorespiratory fitness was 0.58 (95% CI: 0.41–0.81, I^2^ = 0%, p_heterogeneity_ = 0.65) and per 5 METs increase in cardiorespiratory fitness was 0.49 (95% CI: 0.33–0.73, I^2^ = 0%, p_heterogeneity_ = 0.82) (Fig. 4).

There was only one study each reporting on vigorous physical activity [33] and occupational physical activity [34] and risk of sudden cardiac death, thus meta-analyses were not possible for these exposures. The study on vigorous physical activity found no statistically significant association and the study on occupational physical activity found a significant inverse association (Table 1).

### Subgroup analyses, study quality and sensitivity analyses

There were inverse associations between leisure-time physical activity and sudden cardiac death in all subgroup analyses defined by sex, duration of follow-up, year of recruitment, geographic location, number of cases, study quality and adjustment for confounding factors and potentially intermediate factors (including age, family history of sudden cardiac death, family history of myocardial infarction, BMI, smoking, diabetes mellitus, hypertension, systolic blood pressure, cholesterol) (Table 2). With meta-regression analyses there was no evidence that the results differed between these subgroups and there was also no heterogeneity within all the subgroup analyses (Table 2). The mean (median) study quality based on the Newcastle-Ottawa scale was 7.3 (8.0) out of a total of 9 points (Supplementary Table 3). The main contributors to a lower than optimal study quality score were lack of validation or reporting of validation of the physical activity assessment method, lack of exclusion of participants with prevalent cardiovascular disease at baseline, and lack of reporting on loss to follow-up (Supplementary Table 3).

**Table 2:** Subgroup analyses of physical activity and sudden cardiac death

		Physical activity and sudden cardiac death				
		*n*	Relative risk (95% CI)	*I*^*2*^ (%)	*P*_h_^1^	*P*_h_^2^
All studies		8	0.52 (0.45–0.60)	0	0.72	
Sex						
Men		2	0.55 (0.34–0.90)	46.0	0.17	0.65/ 0.77^3^
Women		1	0.47 (0.30–0.72)			
Men, women		5	0.51 (0.43–0.61)	0	0.72	
Follow-up						
< 10 years		4	0.49 (0.39–0.62)	11.3	0.34	0.53
≥ 10 years		4	0.54 (0.45–0.66)	0	0.87	
Recruitment year						
1951–1979		3	0.57 (0.41–0.79)	0.3	0.37	0.27
1980–1999		3	0.55 (0.45–0.67)	0	0.74	
2000–2018		2	0.44 (0.33–0.59)	0	0.82	
Geographic location						
Europe		6	0.50 (0.43–0.60)	0	0.75	0.51
America		2	0.57 (0.40–0.80)	27.3	0.24	
Asia		0				
Number of sudden cardiac deaths						
< 125		4	0.43 (0.33–0.56)	0	0.99	0.13
≥ 125		4	0.57 (0.48–0.68)	0	0.71	
Study quality						
0–3 stars		0				0.39
4–6 stars		2	0.45 (0.31–0.64)	0	0.68	
7–9 stars		6	0.54 (0.46–0.63)	0	0.62	
Adjustment for confounding factors						
Age	Yes	8	0.52 (0.45–0.60)	0	0.72	NC
	No	0				
Family history of sudden cardiac death	Yes	1	0.43 (0.08–1.42)			0.80
	No	7	0.52 (0.45–0.60)	0	0.61	
Family history of myocardial infarction	Yes	2	0.47 (0.31–0.71)	0	0.91	0.60
	No	6	0.53 (0.45–0.62)	0	0.52	
Body mass index	Yes	5	0.51 (0.43–0.61)	0	0.72	0.82
	No	3	0.53 (0.39–0.71)	16.1	0.30	
Smoking	Yes	6	0.54 (0.46–0.63)	0	0.62	0.39
	No	2	0.45 (0.31–0.64)	0	0.68	
Alcohol	Yes	0				NC
	No	8	0.52 (0.45–0.60)	0	0.72	
Diabetes mellitus	Yes	6	0.51 (0.43–0.60)	0	0.82	0.53
	No	2	0.55 (0.34–0.90)	46.0	0.17	
Hypertension	Yes	1	0.47 (0.30–0.72)			0.65
	No	7	0.53 (0.45–0.62)	0	0.64	
Systolic blood pressure	Yes	5	0.51 (0.43–0.61)	0	0.72	0.82
	No	3	0.53 (0.39–0.71)	16.1	0.30	
Blood cholesterol	Yes	7	0.53 (0.45–0.62)	0	0.70	0.43
	No	1	0.40 (0.20–0.70)			

*n* denotes the number of studies

^1^P for heterogeneity within each subgroup

^2^ P for heterogeneity between subgroups with meta-regression analysis

^3^ P for heterogeneity between men and women (excluding studies with both genders) with meta-regression analysis

NC, not calculable because no studies were present in one of the subgroups

In a sensitivity analysis the data from the Multiple Risk Factor Intervention Trial [20] were excluded because the selected participants were at high risk and not representative of the whole population, however, the results remained similar with a summary RR of 0.50 (95% CI: 0.43–0.59, I^2^ = 0%, p_heterogeneity_ = 0.84).

## Discussion

This meta-analysis, which to our knowledge is the first meta-analysis of population-based prospective studies of physical activity and risk of sudden cardiac death, we found that participants reporting the highest level of physical activity had approximately half the risk of sudden cardiac death compared to those with the lowest level of activity. In the dose-response analysis there was a 32% reduction in risk of sudden cardiac death per 20 MET-hours/week, however, the association appeared to be flat above a physical activity level of 20–25 MET-hours/week. There was also a 42% reduction in risk of sudden cardiac death for the highest vs. lowest level of cardiorespiratory fitness, but this finding was limited by being based on only two studies. These results are consistent with many other studies which have shown that physical activity reduces the risk of other cardiovascular outcomes, such as ischemic heart disease [16], stroke [16], and heart failure [35, 36].

As with any meta-analysis of published studies this analysis also has some limitations. Persons who are physically active may have a healthier overall lifestyle with less obesity, lower prevalence of smoking, a healthier diet, and lower prevalence of other risk factors than physically inactive people. It is possible that these factors could have confounded the association between physical activity and sudden cardiac death. However, the inverse association between physical activity and sudden cardiac death persisted across a number of subgroup analyses with adjustment for BMI, smoking, diabetes mellitus, hypertension, systolic blood pressure and blood cholesterol, suggesting an association independent of these risk factors. Although heterogeneity between studies often is observed in meta-analyses because of differences in study design, duration of follow-up, and adjustments for confounding factors, there was no heterogeneity between the studies in this analysis. In addition, publication bias can affect the results of meta-analyses of published studies, however, because of the limited number of studies we did not test for publication bias. Because of limited data on subtypes of activity we were not able to investigate whether specific subtypes of physical activity were particularly beneficial. In addition, because of the way the data were reported by some of the original studies (e.g. < 3 categories of activity) we were not able to include all the available studies in the dose-response analysis. This is a recurring problem with studies on physical activity and point to a need for more standardized reporting of physical activity in epidemiological studies. Any future studies could help move the field forward by reporting results both in hours/week and MET-hours/week and provide results for at least three categories of activity, which will allow for inclusion in linear and nonlinear dose-response analyses. Physical activity was self-reported across studies thus some measurement error and misclassification of the exposure is likely to have occurred, however, because of the prospective design of the included studies, measurement errors would most likely have attenuated the observed associations. Only two studies specifically stated that the physical activity questionnaires had been validated [9, 32]. The remaining studies only stated that they used a physical activity questionnaire or did not describe the physical activity assessment or whether it had been validated, possibly because physical activity was only one of many risk factors that were examined in relation to sudden cardiac death. In addition, most of the included studies may not have had repeated measurements of physical activity during follow-up, which could lead to regression dilution bias, but this would also most likely have attenuated the strength of the associations. Several of the included studies recruited the participants several decades ago when rates of cardiovascular disease were much higher than currently and it is possible that associations may be different when absolute rates are lower as in more recent years. However, there was no heterogeneity between subgroups when the studies were stratified by the year of recruitment and the summary estimates were similar in these subgroup analyses.

Several mechanistic pathways could explain a reduced risk of sudden cardiac death among physically active persons. Physical activity is important for reducing metabolic risk factors including overweight, obesity and weight gain over time [37], hypertension [38–40], elevated heart rate [41], and high cholesterol [40, 42] as well as the risk of diabetes [43], coronary heart disease [16], and heart failure [35, 36], risk factors that are associated with increased risk of sudden cardiac death [9, 11–14, 19, 44]. However, there was little difference between the overall summary estimate and the subgroup analyses of studies that adjusted for diabetes, cholesterol and systolic blood pressure, suggesting that the association is largely independent of these risk factors. Physical activity also has a favourable effect on myocardial oxygen demand and is likely to increase the absolute exercise intensity that is needed for ischemia to occur in a person with existing coronary stenosis [45]. Coronary heart disease is one of the strongest risk factors for sudden cardiac death and studies have reported a 3–5 fold increase in risk of sudden cardiac death among coronary heart disease patients [12, 19, 46]. Interestingly, one study found that physical activity was much more strongly associated with reduced risk of sudden cardiac death among participants with no pre-existing ischemic heart disease compared to participants with pre-existing ischemic heart disease, with reported relative risks of 0.3 and 0.8 for the two subgroups, respectively [19]. This might suggest that reduced risk of ischemic heart disease could be an important pathway through which physical activity reduces the risk of sudden cardiac death. However, further studies with more direct testing of mediation would be needed before conclusions can be made with regard to the potential underlying mechanisms.

Strengths of the present meta-analysis include 1) inclusion of only cohort study designs with reduced potential for recall and selection bias, 2) the detailed subgroup and sensitivity analyses which were consistent with the overall results and which also showed little evidence of heterogeneity, 3) the high study quality of the included studies, and 4) the large sample size providing a robust estimate of the association between physical activity and risk of sudden cardiac death. These results have important public health implications because sudden cardiac death is the first manifestation of heart disease in approximately half of all cases and in most cases there are no warning symptoms. Therefore primary prevention by increasing physical activity may be a promising avenue for reducing the public health burden of sudden cardiac death. A complexity of the available data is that vigorous physical activity or physical exertion in some cases can act as a trigger for cardiac arrest and sudden cardiac death, particularly among persons with already established cardiovascular disease. This is sometimes also observed in younger athletes [47, 48], often because of already existing disease including arrhythmias, cardiomyopathies, congenital anomalies, and valve disorders. However, the incidence of sudden cardiac death is much lower at these younger ages than in middle age, thus any adverse effect of increased physical activity will be low at the population level compared to the adverse effect of low physical activity on risk of sudden cardiac death in middle-aged populations. Nevertheless, it will be important to identify those individuals with pre-disposing conditions that may be at risk from increased physical activity so the combination of a high-risk strategy and population-wide strategies may be most attractive. In addition, further studies are needed to clarify which types and intensities of physical activity may be most beneficial for preventing sudden cardiac death. At the population-level physical activity remains an important modifiable risk factor for sudden cardiac death as well as a protective factor for many other diseases including cardiovascular diseases [16, 35, 36], type 2 diabetes [43], multiple cancers [49], several other diseases [50–54], and premature mortality [55]. Therefore the current results underscore the importance of promoting physical activity for the primary prevention of sudden cardiac death as well as for overall health.

## Conclusion

The current meta-analysis of population-based prospective studies suggests that physical activity may reduce the risk of sudden cardiac death by almost 50% in the general population. Further studies are needed to clarify whether specific subtypes or intensities of physical activity are more beneficial than others and to clarify the dose-response relationship.

## Supplementary information

**Additional file 1.** Webappendix_Physical activity and SCE_09.04.2020_r2. Contains: Search strategy, list of excluded studies, supplementary tables of results from nonlinear dose-response analyses, study quality assessment, supplementary figures with influence analyses and sensitivity analyses.

## Data Availability

The data used are available in the original articles included in the meta-analysis and are described in Table 1.

## Abbreviations

BMI: Body mass index
CI: Confidence interval
kg: Kilogram
MET: Metabolic equivalent task
ml: Milliliter
min: Minutes
O_2_: Oxygen
RR: Relative risk
WHO: World Health Organization
