# Supplementary material for: Physical activity and the risk of sudden cardiac death: a systematic review and meta-analysis of prospective studies
Source: BMC Cardiovasc Disord. 2020 Jul 6;20:318. doi: 10.1186/s12872-020-01531-z (PMC7336483; doi:10.1186/s12872-020-01531-z)

Supplementary Text. Search terms in PubMed

(((("physical activity" OR physical activity[MeSH] OR exercise OR exercise[MeSH] OR sports OR sports[MeSH] OR walking OR walking[MeSH] OR biking OR bicycling OR bicycling[MeSH] OR running OR running[MeSH] OR fitness OR "exercise test" OR exercise test[MeSH] OR inactivity OR sedentary)) AND ("sudden cardiac death" OR sudden cardiac death[MeSH] OR "cardiac arrest" OR cardiac arrest[MeSH]))) AND ("case-control" OR cohort OR prospective OR longitudinal OR retrospective OR "follow-up" OR "cross-sectional" OR "hazard ratio" OR "hazard ratios" OR "relative risk" OR "relative risks" OR "incidence rate ratio" OR "incidence rate ratios" OR "odds ratio" OR odds ratios OR incidence)

Search terms in Embase

(physical activity OR physical activity/ OR exercise OR exercise/ OR sports OR sports/ OR walking OR walking/ OR biking OR biking/ OR bicycling OR bicycling/ OR running OR running/ OR fitness OR fitness/ OR exercise test OR exercise test/ OR inactivity OR inactivity/ OR sedentary OR sedentary/) AND (sudden cardiac death OR cardiac arrest OR sudden cardiac death/ OR cardiac arrest/) AND (case-control OR cohort OR prospective OR longitudinal OR retrospective OR follow-up OR cross-sectional OR hazard ratio OR hazard ratios OR relative risk OR relative risks OR incidence rate ratio OR incidence rate ratios OR odds ratio OR odds ratios OR incidence)

Supplementary Table 1: List of excluded studies and exclusion reasons

Exclusion reason	Reference number
Abstract	(1-9)
Athletes	(10-13)
Case-control study	(14-18)
Case only study	(19)
Duplicate	(20-26)
Editorial, comment	(27;28)
No risk estimate	(29)
Not relevant data	(30;31)
Not relevant exposure	(32)
Not relevant outcome	(33;34)
Patient population	(35-42)
Review	(43-59)
Survival after cardiac arrest	(60;61)
Unspecific outcome	(62)

Reference List

(1) Hamilton A, Moore MJ, Cairns KJ, Adgey AAJ, Kee F. Risk factors in patients with an out-of-hospital cardiac arrest. European Heart Journal Conference: European Society of Cardiology, ESC Congress 2009;(var.pagings):September.

(2) Deo R, Vittinghoff E, Lin F, Tseng ZH, Hulley SB, Shlipak MG. Risk factors for sudden cardiac death in women with coronary disease. Heart Rhythm Conference: 31st Annual Scientific Sessions of the Heart Rhythm Society, Heart Rhythm 2010;(var.pagings):May.

(3) Hagnas M, Kurl S, Rauramaa R, Makikallio TH, Laukkanen JA. Sudden cardiac death risk assessment, combining cardiorespiratory fitness with cardiovascular risk factors. European Heart Journal Conference: ESC Congress 2012;(var.pagings):August.

(4) Hagnas MJ, Sudhir S, Makikallio T, Rauramaa R, Laukkanen JA. High leisure-time physical activity reduces the risk of sudden cardiac death among men with low cardiorespiratory fitness. European Heart Journal Conference: European Society of Cardiology, ESC Congress 2013;(var.pagings):August.

(5) Hagnas MJ, Lakka TA, Kurl S, Makikallio TH, Savonen K, Rauramaa R, Laukkanen JA. Cardiorespiratory fitness modifies the association between leisure-time physical activity and the risk of sudden cardiac death among middle-aged men. Circulation Conference: American Heart Association's 2015;(var.pagings):10.

(6) Kunutsor SK, Khan H, Zaccardi F, Rauramaa R, Laukkanen JA. Oxygen uptake at aerobic thresholds during exercise testing, cardiovascular and all-cause mortality outcomes. Circulation Conference: American Heart Association's 2016;(Supplement 1):November.

(7) Jae SY, Kurl S, Zaccardi F, Willeit P, Kunutsor S, Khan H, Franklin BA, Laukkanen JA. Fitness, body mass index and the risk of sudden cardiac death in middle-aged men: The Kuopio Ischemic heart disease study. European Heart Journal Conference: European Society of Cardiology, ESC Congress 2017;(Supplement 1):August.

(8) Park YA, Yang PS, Kim TH, Uhm JS, Kim JY, Pak HN, Lee MH, Joung B. The impact of regular exercise in cardiovascular mortality and sudden cardiac arrest: A nationwide cohort study. Europace Conference: European Heart Rhythm Association EUROPACE-CARDIOSTIM 2017;(Supplement 3):June.

(9) Park YA, Yang PS, Kim TH, Uhm JS, Sung JH, Kim JY, Pak HN, Lee MH, Joung B. The impact of exercise volume on all-cause mortality, cardiovascular mortality and sudden cardiac arrest: A nationwide cohort study. European Heart Journal Conference: European Society of Cardiology, ESC Congress 2017;(Supplement 1):August.

(10) Risgaard B, Winkel BG, Jabbari R, Glinge C, Ingemann-Hansen O, Thomsen JL, Ottesen GL, Haunso S, Holst AG, Tfelt-Hansen J. Sports-related sudden cardiac death in a competitive and a noncompetitive athlete population aged 12 to 49 years: data from an unselected nationwide study in Denmark. Heart Rhythm 2014 Oct;11(10):1673-81.

(11) Grabs V, Peres T, Zelger O, Haller B, Pressler A, Braun S, Halle M, Scherr J. Decreased prevalence of cardiac arrhythmias during and after vigorous and prolonged exercise in healthy male marathon runners. Am Heart J 2015 Jul;170(1):149-55.

(12) Burke AP, Farb A, Virmani R, Goodin J, Smialek JE. Sports-related and non-sports-related sudden cardiac death in young adults. Am Heart J 1991 Feb;121(2 Pt 1):568-75.

(13) Mohananey D, Masri A, Desai RM, Dalal S, Phelan D, Kanj M, Wazni O, Griffin BP, Desai MY. Global Incidence of Sports-Related Sudden Cardiac Death. Journal of the American College of Cardiology 69 (21) (pp 2672-2673), 2017;30.

(14) Siscovick DS, Weiss NS, Hallstrom AP, Inui TS, Peterson DR. Physical activity and primary cardiac arrest. JAMA 1982 Dec 17;248(23):3113-7.

(15) Siscovick DS, Weiss NS, Fletcher RH, Schoenbach VJ, Wagner EH. Habitual vigorous exercise and primary cardiac arrest: effect of other risk factors on the relationship. J Chronic Dis 1984;37(8):625-31.

(16) Lemaitre RN, Siscovick DS, Raghunathan TE, Weinmann S, Arbogast P, Lin DY. Leisure-time physical activity and the risk of primary cardiac arrest. Arch Intern Med 1999 Apr 12;159(7):686-90.

(17) Wisten A, Messner T. Young Swedish patients with sudden cardiac death have a lifestyle very similar to a control population. Scand Cardiovasc J 2005 Jul;39(3):137-42.

(18) Mellor G, Raju H, de Noronha SV, Papadakis M, Sharma S, Behr ER, Sheppard MN. Clinical characteristics and circumstances of death in the sudden arrhythmic death syndrome. Circ Arrhythm Electrophysiol 2014 Dec;7(6):1078-83.

(19) Campuzano O, Sanchez-Molero O, Fernandez A, Mademont-Soler I, Coll M, Perez-Serra A, Mates J, Del OB, Pico F, Nogue-Navarro L, Sarquella-Brugada G, Iglesias A, et al. Sudden Arrhythmic Death During Exercise: A Post-Mortem Genetic Analysis. Sports Med 2017 Mar 3.

(20) Shaper AG, Wannamethee G, Macfarlane PW, Walker M. Heart rate, ischaemic heart disease, and sudden cardiac death in middle-aged British men. Br Heart J 1993 Jul;70(1):49-55.

(21) Jouven X, Empana JP, Schwartz PJ, Desnos M, Courbon D, Ducimetiere P. Heart-rate profile during exercise as a predictor of sudden death. N Engl J Med 2005 May 12;352(19):1951-8.

(22) Whang W, Manson JE, Hu FB, Chae CU, Rexrode KM, Willett WC, Stampfer MJ, Albert CM. Physical exertion, exercise, and sudden cardiac death in women. JAMA 2006 Mar 22;295(12):1399-403.

(23) Laukkanen JA, Makikallio TH, Rauramaa R, Kiviniemi V, Ronkainen K, Kurl S. Cardiorespiratory fitness is related to the risk of sudden cardiac death: a population-based follow-up study. J Am Coll Cardiol 2010 Oct 26;56(18):1476-83.

(24) Hagnas MJ, Lakka TA, Kurl S, Rauramaa R, Makikallio TH, Savonen K, Laukkanen JA. Cardiorespiratory fitness and exercise-induced ST segment depression in assessing the risk of sudden cardiac death in men. Heart 2017 Mar;103(5):383-9.

(25) Laukkanen JA, Laukkanen T, Khan H, Babar M, Kunutsor SK. Combined Effect of Sauna Bathing and Cardiorespiratory Fitness on the Risk of Sudden Cardiac Deaths in Caucasian Men: A Long-term Prospective Cohort Study. Prog Cardiovasc Dis 2018 Mar;60(6):635-41.

(26) Hagnas MJ, Lakka TA, Makikallio TH, Kurl S, Savonen K, Rauramaa R, Laukkanen JA. High Leisure-Time Physical Activity Is Associated With Reduced Risk of Sudden Cardiac Death Among Men With Low Cardiorespiratory Fitness. Can J Cardiol 2018 Mar;34(3):288-94.

(27) Jae SY, Kurl S, Laukkanen JA, Franklin BA. Physical activity and cardiorespiratory fitness as underappreciated modulators of obesity-related risk of sudden cardiac death. Heart 101 (10) (pp 822), 2015;01.

(28) Albert CM. Sudden cardiac death risk prediction: Challenging the status quo. Archives of Internal Medicine 171 (19) (pp 1710-1711), 2011;24.

(29) Sherman SE, D'Agostino RB, Cobb JL, Kannel WB. Does exercise reduce mortality rates in the elderly? Experience from the Framingham Heart Study. Am Heart J 1994 Nov;128(5):965-72.

(30) Marijon E, Uy-Evanado A, Teodorescu C, Reinier K, Huertas-Vazquez A, Narayanan K, Jerger K, Mariani R, Stecker EC, Chugh H, Navarro J, Jouven X, et al. Sports-associated sudden cardiac death in middle-aged men: Incidence, characteristics and outcomes. Heart Rhythm Conference: 35th Annual Scientific Sessions of the Heart Rhythm Society, Heart Rhythm 2014;(var.pagings):May.

(31) Durakovic Z, Durakovic MM, Skavic J, Gojanovic MD. Physical activity and sudden cardiac death in elders--a Croatian study. Coll Antropol 2011 Mar;35(1):103-6.

(32) Bertoia ML, Triche EW, Michaud DS, Baylin A, Hogan JW, Neuhouser ML, Tinker LF, Van HL, Waring ME, Li W, Shikany JM, Eaton CB. Mediterranean and Dietary Approaches to Stop Hypertension dietary patterns and risk of sudden cardiac death in postmenopausal women1-3. American Journal of Clinical Nutrition 99 (2) (pp 344-351), 2014;01.

(33) Kubota Y, Iso H, Yamagishi K, Sawada N, Tsugane S. Daily Total Physical Activity and Incident Cardiovascular Disease in Japanese Men and Women: Japan Public Health Center-Based Prospective Study. Circulation 135 (15) (pp 1471-1473), 2017;11.

(34) Engeseth K, Prestgaard EE, Mariampillai JE, Grundvold I, Liestol K, Kjeldsen SE, Bodegard J, Erikssen JE, Gjesdal K, Skretteberg PT. Physical fitness is a modifiable predictor of early cardiovascular death: A 35-year follow-up study of 2014 healthy middle-aged men. European Journal of Preventive Cardiology 25 (15) (pp 1655-1663), 2018;01.

(35) Deo R, Vittinghoff E, Lin F, Tseng ZH, Hulley SB, Shlipak MG. Risk factor and prediction modeling for sudden cardiac death in women with coronary artery disease. Arch Intern Med 2011 Oct 24;171(19):1703-9.

(36) Wicks AF, Lumley T, Lemaitre RN, Sotoodehnia N, Rea TD, McKnight B, Strogatz DS, Bovbjerg VE, Siscovick DS. Major life events as potential triggers of sudden cardiac arrest. Epidemiology 2012 May;23(3):482-5.

(37) Ruwald AC, Marcus F, Estes NA, III, Link M, McNitt S, Polonsky B, Calkins H, Towbin JA, Moss AJ, Zareba W. Association of competitive and recreational sport participation with cardiac events in patients with arrhythmogenic right ventricular cardiomyopathy: results from the North American multidisciplinary study of arrhythmogenic right ventricular cardiomyopathy. Eur Heart J 2015 Jul 14;36(27):1735-43.

(38) Siscovick DS, Weiss NS, Fletcher RH, Lasky T. The incidence of primary cardiac arrest during vigorous exercise. N Engl J Med 1984 Oct 4;311(14):874-7.

(39) Kiviniemi AM, Lahtinen M, Junttila MJ, Kaariainen M, Huikuri HV, Tulppo MP. Changes in physical activity and risk for cardiac death in stable coronary artery disease. European Heart Journal Conference: European Society of Cardiology, ESC Congress 2017;(Supplement 1):August.

(40) Tulppo MP, Kiviniemi AM, Lahtinen M, Toukola T, Perkiomaki J, Junttila J, Huikuri HV. Physical activity and risk for sudden cardiac death in stable coronary artery disease. Heart Rhythm Conference: 39th Annual Scientific Sessions of the Heart Rhythm Society, Heart Rhythm 2018;(5 Supplement 1):May.

(41) Reddy PR, Reinier K, Singh T, Mariani R, Gunson K, Jui J, Chugh SS. Physical activity as a trigger of sudden cardiac arrest: the Oregon Sudden Unexpected Death Study. Int J Cardiol 2009 Jan 24;131(3):345-9.

(42) Marijon E, Uy-Evanado A, Reinier K, Teodorescu C, Narayanan K, Jouven X, Gunson K, Jui J, Chugh SS. Sudden cardiac arrest during sports activity in middle age. Circulation 2015 Apr 21;131(16):1384-91.

(43) Kohl HW, III, Powell KE, Gordon NF, Blair SN, Paffenbarger RS, Jr. Physical activity, physical fitness, and sudden cardiac death. Epidemiol Rev 1992;14:37-58.

(44) Mittleman MA, Siscovick DS. Physical exertion as a trigger of myocardial infarction and sudden cardiac death. Cardiol Clin 1996 May;14(2):263-70.

(45) Siscovick DS. Exercise and its role in sudden cardiac death. Cardiol Clin 1997 Aug;15(3):467-72.

(46) Muller-Nordhorn J, Arntz H-R, Lowel H, Willich SN. The epidemiology of sudden cardiac death. Herzschrittmachertherapie und Elektrophysiologie 12 (1) (pp 3-8), 2001;2001.

(47) Melzer K, Kayser B, Pichard C. Physical activity: the health benefits outweigh the risks. Curr Opin Clin Nutr Metab Care 2004 Nov;7(6):641-7.

(48) Franklin BA. Cardiovascular events associated with exercise: The risk-protection paradox. Journal of Cardiopulmonary Rehabilitation 25 (4) (pp 189-197), 2005;July/August.

(49) Corrado D, Migliore F, Basso C, Thiene G. Exercise and the risk of sudden cardiac death. Herz 2006 Sep;31(6):553-8.

(50) Corrado D, Basso C, Schiavon M, Thiene G. Does sports activity enhance the risk of sudden cardiac death? J Cardiovasc Med (Hagerstown) 2006 Apr;7(4):228-33.

(51) Dahabreh IJ, Paulus JK. Association of episodic physical and sexual activity with triggering of acute cardiac events: systematic review and meta-analysis. JAMA 2011 Mar 23;305(12):1225-33.

(52) Mahmoud KD, de Smet BJGL, Zijlstra F, Rihal CS, Holmes DR. Sudden Cardiac Death: Epidemiology, Circadian Variation, and Triggers. Current Problems in Cardiology 36 (2) (pp 56-80), 2011;February.

(53) D'Silva A, Sharma S. Exercise, the athlete's heart, and sudden cardiac death. Phys Sportsmed 2014 May;42(2):100-13.

(54) Dhutia H, Sharma S. Playing it safe: exercise and cardiovascular health. Practitioner 2015 Oct;259(1786):15-20, 2.

(55) Chugh SS, Weiss JB. Sudden cardiac death in the older athlete. J Am Coll Cardiol 2015 Feb 10;65(5):493-502.

(56) Waite O, Smith A, Madge L, Spring H, Noret N. Sudden cardiac death in marathons: a systematic review. Phys Sportsmed 2016;44(1):79-84.

(57) Merghani A, Malhotra A, Sharma S. The U-shaped relationship between exercise and cardiac morbidity. Trends Cardiovasc Med 2016 Apr;26(3):232-40.

(58) Risgaard B. Sudden cardiac death: A nationwide cohort study among the young. Danish Medical Journal 63 (12) (no pagination), 2016;5321.

(59) Asif IM, Harmon KG. Incidence and Etiology of Sudden Cardiac Death: New Updates for Athletic Departments. Sports Health 2017 May;9(3):268-79.

(60) Torell MF, Stromsoe A, Zagerholm E, Herlitz J, Claesson A, Svensson L, Borjesson M. Higher survival rates in exercise-related out-of-hospital cardiac arrests, compared to non-exercise-related - a study from the Swedish Register of Cardiopulmonary Resuscitation. Eur J Prev Cardiol 2017 Oct;24(15):1673-9.

(61) Ro YS, Shin SD, Song KJ, Hong KJ, Ahn KO. Association of Exercise and Metabolic Equivalent of Task (MET) Score with Survival Outcomes after Out-of-Hospital Cardiac Arrest of Young and Middle Age. Resuscitation 2017 Jun;115:44-51.

(62) Andersen K, Rasmussen F, Held C, Neovius M, Tynelius P, Sundstrom J. Exercise capacity and muscle strength and risk of vascular disease and arrhythmia in 1.1 million young Swedish men: cohort study. BMJ 2015 Sep 16;351:h4543.

Supplementary Table 2. Relative risks and 95% confidence intervals from the nonlinear analysis of the association between leisure-time physical activity and sudden cardiac death

MET-hours/week	RRs (95% CIs)	RRs (95% CIs) sensitivity analysis including Hamer et al, 2018
0	1.00	1.00
5	0.79 (0.64-0.99)	0.84 (0.70-1.01)
10	0.67 (0.47-0.95)	0.73 (0.54-0.98)
15	0.61 (0.42-0.88)	0.66 (0.47-0.91)
20	0.60 (0.44-0.81)	0.62 (0.47-0.82)
25	0.60 (0.47-0.77)	0.60 (0.49-0.75)
30	0.62 (0.48-0.80)	0.60 (0.47-0.77)
35	0.64 (0.44-0.92)	0.61 (0.41-0.92)
40	0.66 (0.40-1.08)	0.63 (0.34-1.17)
$P_{nonlinearity}$	0.18	0.35

Supplementary Table 3. Study quality of included studies

Author, publication year	Representative-ness	Selection of non-exposed cohort	Exposure ascertainment	Demonstration of outcome not present at start	Adjustment for one confounder	Adjustment for a second confounder	Assessment of outcome	Long enough follow-up	Loss to follow-up	Total
Leon AS et al, 1987, USA	1	1	1	0	1	1	1	1	0	7
Wannamethee G et al, 1995, United Kingdom	1	1	0	1	1	0	1	1	0	6
Jouven X et al, 2001, France	1	1	0	0	1	1	1	1	1	7
Chiuve SE et al, 2011, USA	0	1	1	0	1	1	1	1	0	6
Lahtinen AM et al, 2012, Finland, FINRISK 1992	1	1	0	1	1	1	1	1	1	8
Lahtinen AM et al, 2012, Finland, FINRISK 1997	1	1	0	1	1	1	1	1	1	8
Lahtinen AM et al, 2012, Finland, FINRISK, 2002	1	1	0	1	1	1	1	1	1	8
Lahtinen AM et al, 2012, Finland, Health 2000	1	1	0	1	1	1	1	1	1	8
Hamer M et al, 2018, United Kingdom	1	1	1	0	1	1	1	1	1	8

Supplementary Figure 1. Influence analysis of leisure-time physical activity and sudden cardiac death

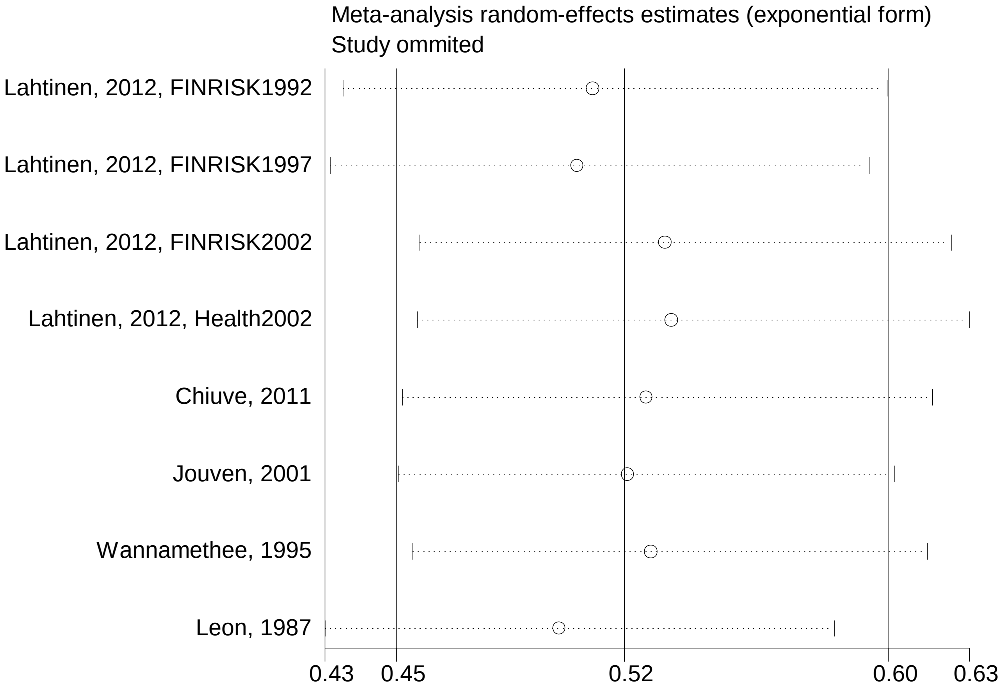

Study omitted	e^coef.	[95% Conf.	Interval]
Lahtinen, 2012, FINRISK1992	0.51020825	0.4319464	0.60264993
Lahtinen, 2012, FINRISK1997	0.50550044	0.42799681	0.59703869
Lahtinen, 2012, FINRISK2002	0.53300029	0.45604306	0.62294406
Lahtinen, 2012, Health2002	0.53490186	0.45522401	0.62852567
Chiuve, 2011	0.52722019	0.45057067	0.61690909
Jouven, 2001	0.521415	0.44938955	0.60498416
Wannamethee, 1995	0.5284946	0.4538939	0.61535639
Leon, 1987	0.49985841	0.42629701	0.58611345
Combined	0.52035358	0.44882823	0.60327722

Supplementary Figure 2. Leisure-time physical activity and sudden cardiac death, sensitivity analyses including the study by Hamer et al, 2018 (which included a combined outcome of sudden cardiac death and arrhythmia)

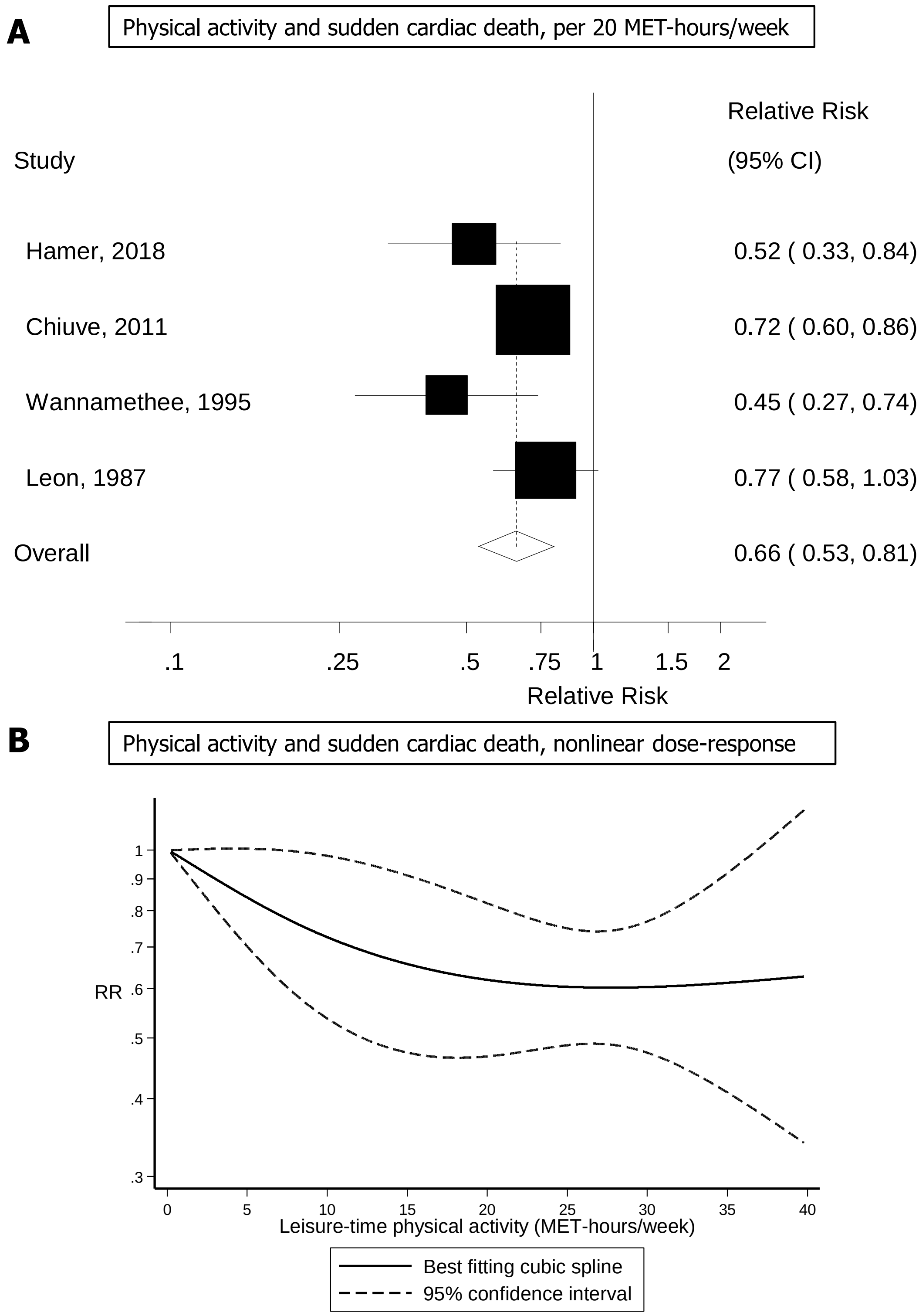

Supplement: Supplementary file 1 — Additional file 1. Webappendix_Physical activity and SCE_09.04.2020_r2. Contains: Search strategy, list of excluded studies, supplementary tables of results from nonlinear dose-response analyses, study quality assessment, supplementary figures with influence analyses and sensitivity analyses. [file 12872_2020_1531_MOESM1_ESM.pdf]
